# Microbial Communities in North American Ixodid Ticks of Veterinary and Medical Importance

**DOI:** 10.3389/fvets.2017.00179

**Published:** 2017-10-20

**Authors:** Andrea S. Varela-Stokes, Si Hong Park, Sun Ae Kim, Steven C. Ricke

**Affiliations:** ^1^Department of Basic Sciences, College of Veterinary Medicine, Mississippi State University, Starkville, MS, United States; ^2^Department of Food Science, Center for Food Safety, University of Arkansas, Fayetteville, AR, United States

**Keywords:** tick vectors, microbiome, next-generation sequencing, pathogens, endosymbionts

## Abstract

Interest in microbial communities, or microbiota, of blood-feeding arthropods such as ticks (order Parasitiformes, suborder Ixodida) is increasing. Studies on tick microorganisms historically emphasized pathogens of high medical or veterinary importance. Current techniques allow for simultaneous detection of pathogens of interest, non-pathogenic symbionts, like Coxiella-LE and *Francisella-*LE, and microorganisms of unknown pathogenic potential. While each generation of ticks begins with a maternally acquired repertoire of microorganisms, microhabitats off and on vertebrate hosts can alter the microbiome during the life cycle. Further, blood-feeding may allow for horizontal exchange of various pathogenic microbiota that may or may not also be capable of vertical transmission. Thus, the tick microbiome may be in constant flux. The geographical spread of tick vector populations has resulted in a broader appreciation of tick-borne diseases and tick-associated microorganisms. Over the last decade, next-generation sequencing technology targeting the 16S rRNA gene led to documented snapshots of bacterial communities among life stages of laboratory and field-collected ticks, ticks in various feeding states, and tick tissues. Characterizing tick bacterial communities at population and individual tissue levels may lead to identification of markers for pathogen maintenance, and thus, indicators of disease “potential” rather than disease state. Defining the role of microbiota within the tick may lead to novel control measures targeting tick-bacterial interactions. Here, we review our current understanding of microbial communities for some vectors in the family Ixodidae (hard ticks) in North America, and interpret published findings for audiences in veterinary and medical fields with an appreciation of tick-borne disease.

## Ticks as Obligate Parasites: Implications for the Tick Microbiome

Ticks represent a unique group of hematophagous ectoparasites capable of transmitting the greatest variety of microorganisms to vertebrate hosts (1). With few exceptions, motile life stages for all three tick families (Ixodidae, Argasidae, and Nuttaliellidae) require a blood meal. The significance of ticks as vectors is not new. Ticks were the first arthropod to be associated with transmission of a disease agent. In the late 1800s, Smith and Kilbourne established *Rhipicephalus (Boophilus)* ticks as the vector for (Texas) cattle fever, caused by *Babesia bigemina* (2). After that discovery, the ability of ticks to acquire, maintain and transmit pathogens became a significant and productive area of scientific research. Historically, tick-borne diseases were described first and then the etiologic agent pursued, as with Texas cattle fever. However, in some cases, tick-associated microorganisms of unknown pathogenicity were identified first, and an association with human or animal disease found later. Better diagnostic assays and heightened recognition of tick-borne diseases contributed to re-descriptions of tick-associated microorganisms. The spotted fever group rickettsia, *Rickettsia parkeri*, was recovered from *Amblyomma maculatum* in Texas approximately 60 years prior to the index case, reported in 2004 (3). Additionally, the spirochete, *Borrelia miyamotoi*, first reported from *Ixodes persucatus* in Japan, was considered non-pathogenic until the first human cases 15 years later (4, 5). New reports of emerging pathogenic tick-borne bacteria, viruses, and protozoa are not rare (6–9). However, most tick-associated microorganisms are not likely pathogens.

There is increasing interest in the tick microbial community, how it may impact transmission and maintenance of pathogens, and how manipulation of the microbial community may serve as an avenue for tick or pathogen control (10). The microhabitat at the surface of a vertebrate host, such as a human, presents a complex source of organisms for ticks to potentially acquire (11). In addition, microorganisms may be exchanged through co-feeding as well as from the external environment, considering ticks spend approximately 90% of their life off the host. Further, the tick itself, through vertical transmission, contributes to the microbiome of its next generation. There are at least ten bacterial genera that are transovarially (vertically) transmitted, including some found only in ticks (e.g., *Coxiella-*LE, *Francisella-*LE, and *Midichloria*), and some common among various arthropods (e.g., *Wolbachia* and *Arsenophonus*) (12). Hawlena et al. found that the microbiomes of the ticks, *Dermacentor variabilis* and *I. scapularis*, were most affected by arthropod-related factors, rather than the host or environment (13). Not surprisingly, the preponderance of *Francisella-*LE and *Arsenophonus* genera in *D. variabilis*, and *Rickettsia* in *I. scapularis*, in their study likely depended more on vertical transmission. A combination of factors is likely, depending on the tick-associated microorganism.

With the development of next-generation sequencing technology, the number of taxa detected in ticks has risen sharply. Enthusiasm over tick microbiome studies has generated a fresh wave of hypotheses to test and has led to a variety of methodologies to be considered in future studies. Microbiome sequencing based on the 16S rRNA gene continues to be widely utilized, but reliable sequencing data requires high quality DNA of sufficient quantity. Microbial genomic DNA extraction methods may vary based on the sample matrix and target DNA (prokaryotic or eukaryotic). Different bacterial cell wall structures may result in bacterial species bias in an extract, depending on genomic DNA recovery method. Yuan et al. tested six DNA extraction methods for human associated sample microbiome sequencing (14). Each method exhibited different bacterial genomic DNA recovery, with bead beating combined with mutanolysin exhibiting high cell lysis efficiency. Kit contaminants must also be considered (15). As tick microbiome analyses continue, the utility of archived extracts, samples that were not sufficiently cleaned prior to extraction, or extracts not enriched for microorganisms may improve if environmental contaminants can be distinguished from true symbionts, and samples are processed accordingly to minimize loss of these symbionts. In recognizing the limitations of published data, the scientific community and readership can better understand the biological significance of the tick microbiome.

## Microbiota Associated with Common Tick Vectors in North America

**Figure 1:**
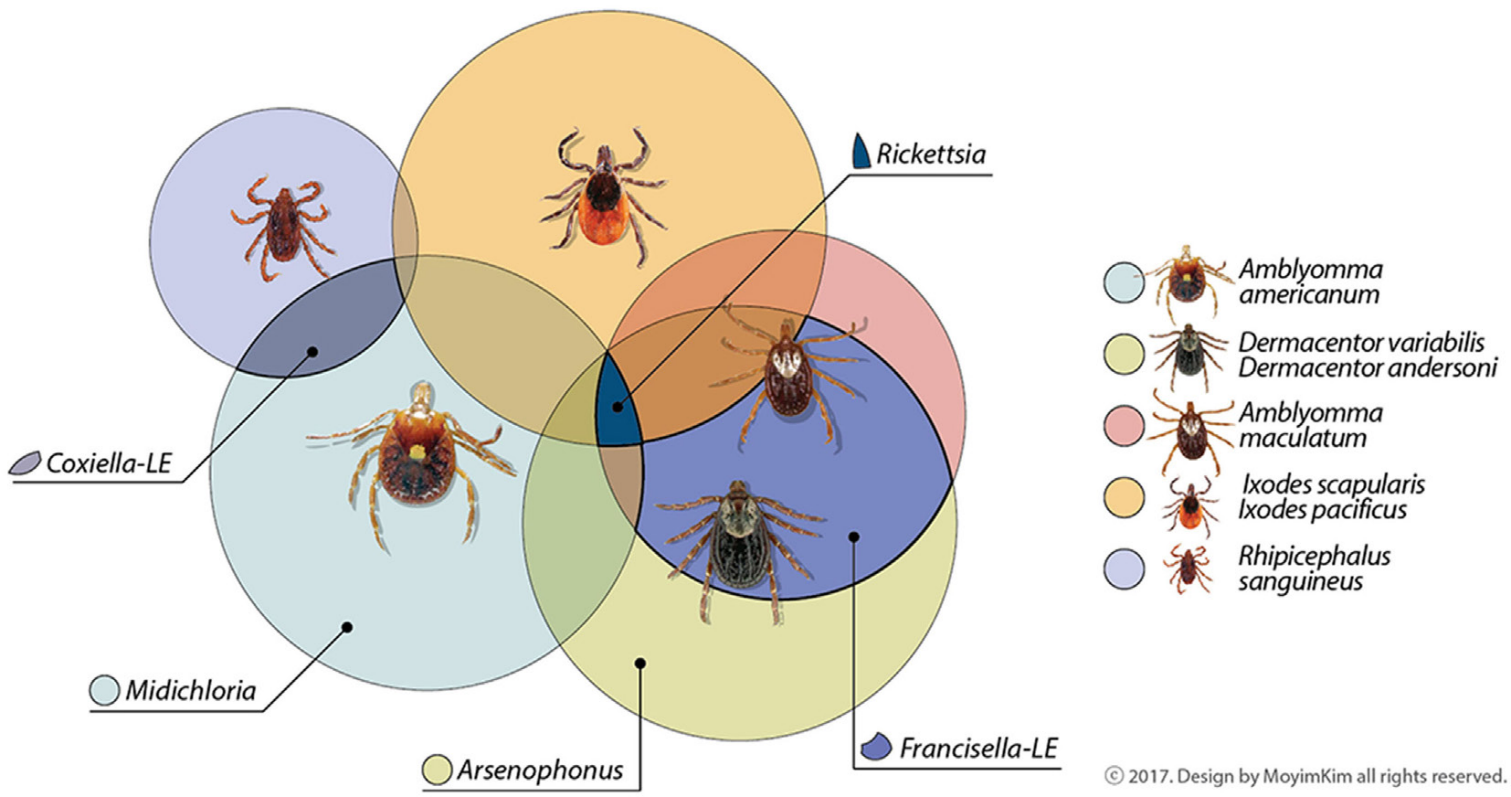
Common maternally inherited symbionts detected in North American tick vectors belong to the Proteobacteria. Genera are in families *Rickettsiacea* (*Rickettsia*), *Coxiellaceae* (*Coxiella-*LE), *Francisellaceae* (*Francisella-*LE), *Midichloriacea* (*Midichloria*), and *Enterobacteriaceae* (*Arsenophonus*). Some symbionts not depicted here as shared among tick vectors have been occasionally reported in the literature or may be reported as common symbionts in the future as data increase. Other areas of overlap may also occur among tick symbionts; these are suggested by unlabeled areas.

Microorganisms living in close relationship with ticks are symbionts and can be categorized as obligate or facultative based on the potential for vertical or horizontal transmission and requirement for survival and reproduction, though these characteristics can be difficult to measure (16). Metabolic contributions from arthropod symbionts were recently reviewed (17). Co-infections with tick-borne pathogens, non-pathogens or uncharacterized bacteria are also documented, as well as evidence that the community of symbionts may influence maintenance and transmission of known pathogens (18). The abundance of microorganisms identified and novel associations uncovered continue to generate questions. Here, we highlight bacteria consistently dominating microbiome catalogs of ticks, focusing on some tick species of vector importance in North America. Obligate symbionts are summarized in Figure 1. For further reading, we suggest Narasimhan and Fikrig (10), Bonnet et al. (12), and Duron et al. (19).

The phylum Proteobacteria comprises the majority of bacterial species detected in ticks (20). Obligate symbionts that predominate include the genera *Coxiella*-LE in *Amblyomma americanum* and several *Rhipicephalus* spp., *Francisella-LE* in *D. variabilis*, and *A. maculatum, Rickettsia* in *I. scapularis*, and *Midichloria* in *A. americanum* (21–23). *Wolbachia* endosymbionts, which are commonly found in other arthropods and some nematodes, are generally rare in ticks (24–26). Pathogens, when detected, seem to constitute a lower percentage of the bacterial population. Thus, one might predict that their presence does not impact the remaining microbial community. In comparing the community structure of bacteria from pathogen-infected (*Anaplasma* or *Ehrlichia*) and uninfected *A. americanum*, bacterial community structure did not significantly differ (27). Three phyla, Proteobacteria, Bacteriodetes, and Firmicutes, and genera *Coxiella*-LE and *Rickettsia, Flavobacterium*, and *Bacillus*, respectively, were commonly encountered, though there were discrepancies between Illumina sequencing and PCR for *Anaplasma* and *Ehrlichia* (27). In addition, while *Coxiella*-LE was more abundant in female compared to male ticks, the genus exhibited low relative abundance overall, and in comparison to *Rickettsia*. “*Candidatus* Rickettsia amblyommii” (now *Rickettsia amblyommatis* sp. nov.) (28) and “*Candidatus* Midichloria mitochondrii” dominated in *A. americanum* from North Carolina (23). Conversely, bacterial communities in *A. americanum* from Georgia were dominated by *Rickettsia* and *Coxiella*-LE, and exhibited frequent contributions from pathogenic (*Ehrlichia*) and uncharacterized genera (e.g., *Midichloria*) (29). While *Coxiella*-LE may be frequently found in *A. americanum* populations (30), the contribution of this genus to microbial abundance may be minimal. Microbial populations in both colony and field-collected *A. americanum* appear to increase in diversity during tick feeding at the expense of contributions from *Coxiella*-LE (31).

Microbiomes of laboratory-reared and wild ticks are likely influenced by generations of rearing in a specific environment and on specific hosts. Short-term environmental changes in a population do not appear to significantly impact the microbiome. The *Rickettsia* genus is rarely detected in colony-reared *A. americanum* ticks compared to overwhelming infection rates of *R. amblyommatis* sp. nov. (“*Candidatus* R. amblyommii”) in wild *A. americanum* (28, 32–34). In colony-reared *A. americanum* nymphs, there was an overall loss in microbial diversity regardless of whether nymphal ticks molted and aged outdoors or in the laboratory; *Coxiella-*LE was present in all tick groups, though *Rickettsia* was not detected in any group (34). These findings support short-term changes in the environment as having a minimal contribution on microbial communities. A closed related tick, *A. maculatum* shares a significant portion of its geographical range with *A. americanum* in the USA but differs in its phenology, microhabitat, host preferences, and obligate symbionts. Microbiome analyses of *A. maculatum* revealed that salivary glands, but not midgut tissues, of laboratory-reared and field-collected ticks commonly contained an abundance of *Francisella-*LE, while the genus *Rickettsia* was predominantly located in midguts. In addition, bacteria from the family *Enterobacteriacea*, which have not been commonly detected in *A. americanum*, constituted a greater portion of sequencing reads from both collections of ticks (26).

*Ixodes scapularis* has public and veterinary health importance as a vector for agents including *B. burgdorferi, Anaplasma phagocytophilum*, and the protozoan, *Babesia*. The increasing incidence of Powassan virus II (deer tick virus) in New England recently renewed anxiety over this high profile vector (35). Disease prevalence varies geographically, with states in the northeastern USA contributing the majority of case reports for *I. scapularis-*associated diseases such as Lyme disease, in comparison to states in the southern USA within the *I. scapularis* range (36). Geographic variations in microbiota are documented in *I. scapularis* in the Mid Atlantic USA, South Atlantic USA, and New England. *Enterobacteriaceae* were dominant in North Carolina *I. scapularis* populations, whereas *Rickettsia (Rickettsiaceae*) were dominant from populations in other USA states; interestingly, a population of *I. scapularis* with high levels of *Borrelia*, in comparison to known *Borrelia-*endemic sites in New England, was identified in Virginia (37). Overall, male *I. scapularis* demonstrated greater diversity compared to females, an observation noted in other tick microbiome studies (23, 37). In an earlier study, 16S rRNA amplicons from *I. scapularis* were separated by temporal temperature gradient gel electrophoresis and sequenced, revealing the dominance of *Rickettsia* in New York populations (38). Additional genera included not only *Borrelia* and *Anaplasma*, but also *Pseudomonas, Ralstonia* and *Rhodococcus*, which encompass species found in the soil, members of *Enterobacteriaceae*, and *Moraxella*, that are considered commensal species in mucosal membranes.

Deforestation, increased urbanization, warmer winters and longer transitional autumn, and spring seasons will help expand the dissemination of some tick species into more northern geographical regions as well as to higher elevations (39, 40). With this expansion is an increasing opportunity for ticks to contact different microbial communities outside of the microbial consortia encountered in traditional geographical regions. This is important because the vector microbiome may affect vector competence. *Ixodes scapularis* larvae reared under primarily sterile conditions and fed on gentamycin-treated mice underwent an altered microbiome (“dysbiosis”). Larvae demonstrated diminished feeding and *B. burgdorferi* colonization, as well as lower expression of the glycoprotein, peritrophin, suggesting an intimate association between gut microbiome composition and integrity of the peritrophic membrane (41). Whether the overall microbiome, or niche-specific variations in tick tissues are relevant to tick-borne pathogen maintenance and transmission may depend on the pathogen’s natural history, including its ability to be vertically transmitted and its requirement for a vertebrate reservoir. As *B. burgdorferi* does not utilize transovarial transmission for maintenance, efficient acquisition from a reservoir host is likely critical for vector competency. The presence of certain pathogens may also modify tick microbiome composition. Abraham et al. demonstrated that *I. scapularis* infected with *A. phagocytophilum*, are induced to express the *I. scapularis* anti-freeze glycoprotein, resulting in diminished biofilm formation in the tick gut and allowing for *A. phagocytophilum* colonization (42). Dysbiosis here acted to enhance pathogen establishment.

*Dermacentor andersoni* and *D. variabilis* are two key vectors for *Rickettsia rickettsii*, the agent of Rocky Mountain spotted fever. *Dermacentor* spp. are predominantly colonized by *Francisella*-LE, although other arthropod symbiotic genera are common, including *Arsenophonus* and *Rickettsia* (21, 43, 44). In *D. andersoni*, microbial diversity shifted over three generations and could be manipulated with tetracycline imbibed with the blood meal (43). Interestingly, *Acinetobacter* was found to increase in salivary glands over three tick generations, and most significantly in ticks fed on tetracycline-treated calves, at the expense of *Arsenophonus, Rickettsia*, and *Francisella-*LE abundance. *Acinetobacter*, a ubiquitous genus found in soil, water, and in normal animal flora, includes species that are now considered pathogenic; further, multidrug-resistant strains of *A. baumannii* are being increasingly found in hospital settings (45). *Francisella-*LE and *Arsenophonus* were common endosymbionts in *D. variabilis* removed from wild *Peromyscus leucopus* mice collected in Indiana, whereas *Acinetobacter* was not detected (44). *Ixodes scapularis*, also collected from *P. leucopus* in this study, did not have evidence of *Francisella-*LE or *Arsenophonus* but was dominated rather by the *Rickettsia* endosymbiont (44).

*Rhipicephalus sanguineus*, a common tick vector found infesting dogs world-wide, will also attach and transmit disease agents to other hosts, including humans. This is significant considering *R. sanguineus* is responsible for transmission of canine bacterial and protozoan pathogens (e.g., *Ehrlichia canis, Babesia vogeli*, and *Hepatozoon canis*) and was recently implicated as the vector in a Rocky Mountain spotted fever outbreak in Arizona, where both dogs and humans were infected (46). Similar to *A. americanum*, the primary endosymbiont is *Coxiella*-LE (19). However, there is minimal information using next-generation sequencing about additional symbionts in the *R. sanguineus* microbiome. Using PCR and sequencing, other genera detected from *R. sanguineus* outside of North America include *Rickettsia*, a genus most closely related to *Wolbachia*, and *Midichloria* (47–49). An initial assessment of the *Rhipicephalus (Boophilus) microplus* microbial community provides further insight as it demonstrated a preponderance of *Coxiella*-LE associated with female ovaries and eggs (50). *Borrelia, Wolbachia*, and potential environmental contaminants including *Staphylococcus* spp. and *Streptococcus* spp. were also detected (50).

## Blood Feeding in Driving the Tick Microbiome

The host blood meal and the process of imbibing blood induce physiological changes in ticks that appear to affect the tick microbiome. In fact, the vertebrate host species may itself affect the microbiome. For example, nymphs of the Lyme disease vector in the western USA, *I. pacificus*, demonstrated reduced microbiome species richness, replaced by a preponderance of *Rickettsia*, after feeding on the western fence lizard, a host that is refractory to *B. burgdorferi*. In comparison, nymphs that fed on *B. burgdorferi* reservoir hosts, mice, exhibited greater diversity at the expense of *Rickettsia*; they also demonstrated a significant reduction of microbial populations through maturing life stage (51). At a minimum, these data imply a relationship between host blood meal and microbiome diversity. While blood meal causes significant changes within the microenvironment of a tick, the extent to which this affects microbial populations may ultimately depend on microbial adaptability.

As erythrocytes lyse in the tick gut lumen, midgut epithelial cells take up hemoglobin and other proteins through endocytosis, where intracellular digestion occurs. Heme, one of the products of digestion, must be detoxified due to its ability to damage tissues through the production of reactive oxygen species and free radicals (52). Kumar et al. found that experimentally increasing oxidative stress in *A. maculatum*, using RNA interference to silence the catalase gene (*CAT*), did not decrease estimated total bacterial loads from salivary glands and midgut tissues (53). In fact, knockdown of *Cu/Zn-SOD* or *Mn-SOD*, two enzymes that additionally function to decrease oxidative stress, actually increased bacterial load in the midgut with reciprocal effects on bacterial load in the salivary glands; in contrast, copy numbers of the pathogen, *R. parkeri*, decreased in these tissues after *Cu/Zn-SOD* silencing, suggesting a differential effect on different bacterial species (54). The digested blood meal itself may be a direct source of antimicrobial compounds. In midgut extracts from *D. variabilis* that were capillary-fed *B. burgdorferi*, α- and β-chain hemoglobin fragments, as well as host ubiquitin in a complex with ribosomal S30, were associated with inhibition of the non-pathogen, *Micrococcus luteus*. As expected, *B. burgdorferi* spirochetes in midguts of *D. variabilis*, which are incompetent vectors for *B. burgdorferi*, were not viable, though spirochetes remained intact (55). Interestingly, in *I. scapularis*, glutathione peroxidase (GPx/Salp25D), a homolog to the peroxiredoxin antioxidants, has a protective effect on *B. burgdorferi* during acquisition by feeding on an infected host, though no effect on spirochete transmission (20). *Ixodes scapularis* GPx/Salp25D also has an apparent protective effect on the pathogen, *Anaplasma marginale* (56). In the *A. maculatum-Rickettsia* system, the function of Salp25D and other selenoprotein antioxidants is not entirely straightforward. Knockdown of the selenocysteine elongation factor resulted in significantly decreased transcription of various selenoprotein antioxidants, with the exception of *SalpD25* (*GPx*), and resulted in a loss of antioxidant capacity in midgut and salivary gland tissues of field-collected *A. maculatum*. However, while *R. parkeri* levels expectedly diminished in the midgut, levels doubled in salivary glands (57).

There may be interesting metabolic implications with the presence of *Enterobacteriaceae* in ticks, considering normal aerobic respiration by this group is associated with release of reactive oxygen species. Survival mechanisms to withstand the resultant oxidative stress are well-documented in *E. coli* and are conserved in related bacteria, with homologous regulators present in members of the phylum Proteobacteria (58). In field-collected *A. maculatum* removed from hosts 8 days post-infestation, *Enterobacteriaceae* constituted the second most abundant group in the midgut, and was also strongly represented in laboratory-reared *A. maculatum*; *Arsenophonus* was not among the *Enterobacteriaceae* identified (26). *Ixodes scapularis* from North Carolina were dominated by *Enterobacteriaceae*, unlike *I. scapularis* from Virginia, South Carolina, Connecticut, and New York (37). Van Truren et al. also observed an inverse relationship between *Enterobacteriaceae* and the genus *Rickettsia* (family *Rickettsiaceae*) in *Ixodes* spp. populations; *Rickettsia* was rare in North Carolina sites dominated by *Enterobacteriaceae* (37).

Tick feeding is also associated with measurable changes in osmolarity within the gut environment, which in turn may affect microorganisms. In *I. scapularis*, morphology and motility of *B. burgdorferi* spirochetes were altered, and expression of key virulence factors stimulated, in association with fluctuations in osmolarity during tick feeding (59). Finally, once present within the tick, ticks may respond to microorganisms though their innate immune system, consisting of both humoral and cellular mechanisms (60). However, the mechanisms by which the tick immune system impacts the composition of the tick microbiome in the midgut and other tissues are poorly understood and beyond the scope of this review.

## Conclusion

Over the last few decades, the scientific community has witnessed and driven the development of next generation sequencing platforms for microbiome sequencing of the gastrointestinal tracts in humans, animals, and insects. Because ticks are significant vectors associated with emerging pathogens, understanding the contributions of the tick microbiome in tick physiology and pathogen stability may lead to novel approaches to vector and pathogen control. With the tick genome described (61), the opportunity to apply the corresponding transcriptomics and proteomics, as well as metabolomics, may help to uncover some of the answers to the interaction between the tick host and its microbiome, and in turn how these interactions impact the presence of pathogens.

## Author Contributions

Contributed to review of the literature, identification of relevant publications to include, and manuscript preparation; involved in editing drafts prior to submission: AV-S, SP, SK, and SR.

## Conflict of Interest Statement

The authors declare that the research was conducted in the absence of any commercial or financial relationships that could be construed as a potential conflict of interest.
